# Opportunities and challenges for smallholder pig production systems in a mountainous region of Xishuangbanna, Yunnan Province, China

**DOI:** 10.1007/s11250-012-0166-5

**Published:** 2012-05-20

**Authors:** Simon Riedel, Anne Schiborra, Christian Huelsebusch, Mao Huanming, Eva Schlecht

**Affiliations:** 1 Animal Husbandry in the Tropics and Subtropics, University of Kassel and Georg-August-Universität Göttingen, Steinstr 19, 37213 Witzenhausen, Germany; 2 Deutsches Institut für Tropische und Subtropische Landwirtschaft GmbH, Steinstrasse 19, 37213 Witzenhausen, Germany; 3 Faculty of Animal Science and Technology, Yunnan Agricultural University, Kunming, Yunnan 650201 China

**Keywords:** Breeding management, Feeding, Small-scale pig production, South East Asia, Southern China

## Abstract

China’s small-scale pig keepers are the largest community of pork producers worldwide. About 56 % of the world's pigs originate from such systems, each producing 2–5 head per year. This study analyzes pig smallholders in Xishuangbanna, a prefecture of Yunnan Province. Categorical principal component analysis and two-step cluster analysis were used to identify three main production systems: livestock-corn-based (LB; 41 %), rubber based (RB; 39 %), and pig based (PB; 20 %) systems. RB farms earn high income from rubber and fatten cross-bred pigs, often using purchased feeds. PB farms own similar-sized rubber plantations and raise pigs, with fodder mainly being cultivated and collected in the forest. LB farms grow corn, rice, and tea while also raising pigs, fed with collected and cultivated fodder as well. About one third of pigs were marketed (LB, 20 %; RB, 42 %; PB, 25 %), and local pig meat is highly appreciated in the nearby town. High mortality, low reproductive performance, and widespread malnourishment are the systems' main constraints. Basic training in hygiene and reproduction management could significantly increase production; most effective measures would be counterbalancing seasonal malnourishment and exploration of locally available protein feeds. Through support by external expertise, farmers could more effectively trade their pigs at lucrative town markets.

## Introduction

Triggered by China’s tremendous economic development, the country’s livestock sector currently undergoes a massive restructuring. Consumption of meat increased by 230 % between 1995 and 2008 (FAO 2010), with a net import close to zero (0.1 %; Delgado et al. 1999). About 50–80 % of all pigs produced in China originate from smallholder farms (Somwaru et al. 2003; Neo and Chen 2009; State Statistical Bureau 2009) while a smaller, but steadily growing number of pigs are produced in factory-like production systems, often supported by foreign companies and capital (Telegraph 2008; AgFeed 2010; Somwaru et al. 2003). While economic transition of very poor rural inhabitants to poor ones produces new livestock keepers and substitutes emigrating farmers, the ongoing trend towards improved productivity (Delgado et al. 1999) and higher earnings from pig farming keeps the market attractive even for small producers. Food from local sources is preferred by rural (Hu et al. 2004) as well as urban citizens (Shanghai: Goldman 2000; Hong Kong: Goldman et al. 1999).

Taken together, these aspects suggest that smallholder pig producers are, and will continue to be, very important for China’s meat sector. Their support seems advisable from a socio-economic as well as ecological point of view and requires a sound understanding of their production systems. To contribute to this task, the present study analyzed the general characteristics and pig management of smallholder pig farms in mountainous areas of southern Yunnan Province so as to identify support strategies that could assist their economic and ecologically sustainable development.

## Materials and methods

### Location

The study was conducted in Xishuangbanna Prefecture, which is located in China’s utmost southern Province Yunnan. Outliers of the Himalaya make the prefecture montane (450–2,500 m a.s.l.); the climate is humid subtropical with mild to cool winters (18.1–21.7 °C) and hot, humid summers (*Cwa* in: Peel et al. 2007). Most of the annual rainfall (1,200–1,600 mm) is falling during monsoon time (May–October). In higher altitude ranges, temperature drops as low as 4 °C in winter. Of Xishuangbannas' 850,000 inhabitants, 400,000 are living in town-like surroundings. Through the remoteness of the region, cultural as well as natural heritage has been maintained in an extraordinary way and underlies several conventions of protection (Myers et al. 2000).

### Agricultural production

Typical Southeast Asian rice–buffalo integrated farming systems (Devendra and Thomas 2002a) were prevalent for ages in Xishuangbanna until the early 1970s when first rubber tree (*Hevea brasiliensis* Müll. Arg.) plantations were established in the region. Ever since, the plantations expanded massively through continually rising natural rubber prices and a sense of entrepreneurship of the tribal people in the area (Sturgeon 2010). The development had tremendous impact on farmers' socio-economic status but also induced massive degradation of the region’s natural resources (Qiu 2009). As an example, forest areas in Menglun Township of Xishuangbanna declined from 48.7 % to 31.1 % between 1988 and 2006 (Hu et al. 2008). Areas exempted from rubber cultivation by law (e.g., rice terraces are not allowed to be turned into perennial cropping areas) are today planted with tropical fruits. In the uplands (above 1,000 m a.s.l.), the rubber tree cannot be grown due to its sensitivity to lower temperatures (Rao et al. 1998; Guo et al. 2006). There, highly integrated crop-livestock farms, sometimes combined with aquaponics (Yongneng et al. 2006), still persist. Major upland crops are tea, corn, and rice, while fiber-hemp has been introduced through a private company in some villages in 2008.

Due to the focus on latex production, livestock production lost its regional importance. Swamp buffaloes (*Bubalus bubalis carabanesis* L.), formerly kept as transport and draft animals, are largely replaced by small tractors. Most pork and chicken meat offered at local wet markets originates from large-scale farms of 400 km distant Simao town or smaller-scale intensive systems around Jinghong. Local pork, however, still is produced and preferred by farmers and town citizens. The so-called ‘Yunnan short-eared pig’ or ‘Small Ear Pig’ (SMEP; FAO 1984; DAGRIS 2007) is the dominant local pig breed.

### Data collection

Data on rural farming systems were collected in the Naban River National Nature Reserve (NRNNR, 26,000 ha, 24 villages, 450–2,300 m a.s.l.) about 30 km northwest of Jinghong. The policy of the reserve follows the UNESCO “Men-Biosphere” (MEB^1^) approach, and apart from the strictly protected core zone (3,900 ha), land is relatively free of restrictions. The reserve represents a cross-section through the prefecture’s total population and agro-ecological systems, with commercial rubber farms in the valleys and subsistence farming at higher altitudes. A household survey was conducted between January and May 2008. Interviewed households (HH) were selected through stratified random sampling with altitude class and ethnic group serving as strata. Altitude (400 m–1,600 m a.s.l.) was divided into three classes of equal range (400 m): lowland (L; 400–800 m a.s.l.); midland (M; 801–1,200 m a.s.l.), and highland (H; >1,200 m a.s.l.). Distinguished ethnic groups were Han, Dai, Lahu, Hani, and other minor groups. With ethnic groups not all being present at each altitude zone, 13 combinations of altitude × ethnic group were differentiated. Surveying 13–17 farms per strata resulted in 201 recorded households. Socio-economic characteristics, and plant and livestock production details were surveyed; a special focus was set to livestock production. Structured open interviews with key persons or institutions, such as the Agricultural Bureau of Xishuangbanna, governmental veterinarians, livestock product suppliers, as well as group discussions with farmers and local community development authorities provided additional information.

### Data analysis

To identify households with different production systems, a clustering procedure was used. All variables were coded into numbers, whereby scaled variables were kept in their original state, and two-class nominal characteristics (e.g., male/female) were coded into binaries. Each qualitative trait with more than two expressions (such as village, ethnic group) was coded into a nominal categorical scheme where one numeric character represents one trait expression.

Household data with many missing values (seven cases) and with an illogical combination of values (six cases) were excluded, resulting in a data set of 184 HH used for further analysis. All steps of data analysis were performed with PASW 18 (formerly SPSS 18; SPSS 2010).

While good clustering algorithms for either scaled or categorical variables exist, few can handle mixed datasets. Among these are the K-mode (Huang 1998), BIRCH algorithm (Zhang et al. 1997), the Hierarchical Clustering, and the two-step clustering approach developed by SPSS (SPSS 2007). Two-step clustering is a system well-suited for identifying an adequate number of clusters and coping with multi-attributed and multi-distributed datasets while not being sensitive for distribution of variables (Bacher et al. 2004). Two-step clustering was therefore chosen for farm type classification in the present study, applying the subsequent steps of pre-selection of relevant variables and of clustering.

Variables were pre-selected through expert validation (Vyas and Kumaranayake 2006) and Categorical Principal Component Analysis (CatPCA). The following variables were maintained for further analysis: land size (hectares of land cultivated in 2007) of rubber, tea, corn, hemp, and rice. Socio-economic variables were family size (scaled variable), off-farm income (scaled), distance to town (scaled), as well as altitude class, and ethnic group as ordinal categorical variables. Indicators for animal husbandry were numbers of pigs, buffaloes, and chicken, which were converted into Tropical Livestock Units (TLU, animal equivalent of 250 kg live weight; 1 TLU = 0.83 buffalo; 5 pigs; 100 chicken). Indicators for the intensity of pig keeping were *“controlled mating”* and *“use of commercial feedstuffs”* (binary variables). Additionally, each production branch was added as binary code, such as *“has pigs yes/no.”* The variables with an absolute component loading (Eigenvalue) greater than 0.5 were: *keep_sow_yes/no*, *TLU_pig*, *TLU_buffalo*, *size_of_corn_fields*, *altitude_class*, *size_of_tea_fields*, and *size_of_rubber_fields*.

**Fig. 1:**
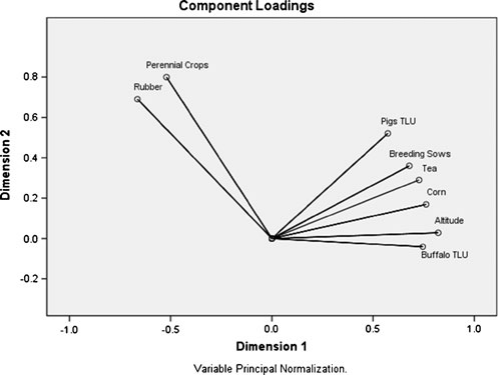
Preliminary outcome of the categorical principal component analysis identifying major variables that characterize smallholder farming systems in the Naban River National Nature Reserve, Xishuangbanna. Distance to center (*x* = *y* = 0) indicates relevance of variable for cluster creation (larger distance = higher relevance), and distance between two variables indicates their degree of correlation (larger distance = lower correlation). Crop areas in hectares; buffaloes and pigs in TLU; breeding sows as binary “yes/no”, altitude (m a.s.l.) in three classes (*1*, 400–800 m a.s.l.; *2*, 801–1,200 m a.s.l.; *3*, >1,200 m a.s.l.)

Figure 1 presents these variables in a two-dimensional chart; a larger distance of a variable to the center indicates a higher significance for cluster building, and a larger distance between two variables indicates a lower correlation between them.

**Fig. 2:**
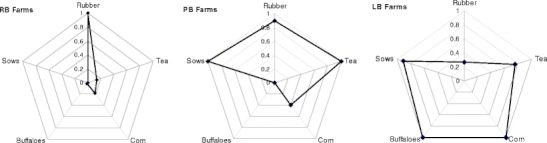
Production diversification of rubber-based (RB), pig-based (PB), and livestock-corn-based (LB) farming systems in the Naban River National Nature Reserve, Xishuangbanna. Each *dot* in the spider web displays the relative importance of one production branch whereby *1* is equivalent to the highest relative possible expression of a trait found in the total population (for example, PB farms, compared with RB and LB farms, have the highest average tea-field size (0.890 ha); their relative-tea-score was set to 1. In comparison, average tea-field size on LB farms is 0.531 ha, which is 60 % of PB tea field size; consequently, their tea-score is 0.6), and a broad web indicates a higher level of diversification

Clustering was then performed to identify the most powerful discriminating variables and appropriate number of classes in order to assign individual farms to the created clusters (Table 1). Starting with the seven most meaningful variables (Fig. 1), the two-step clustering successively eliminated the variables with the lowest impact on cluster creation until a sound equilibrium between the number of variables and the cluster quality (“silhouette measure of cohesion and separation;” SPSS 2007) was found with five variables (*has_buffalo, has_sow, size_of_corn_fields, size_of_rubber_fields, size_of_tea_fields*), leading to a separation of 3 clusters and an average silhouette measure of 0.6 (Fig. 2).

**Table 1:** Cluster-determining variables identified through categorical principal component analysis (CatPCA) and two-step cluster analysis for grouping 184 smallholder farm households (HH) in the Naban River National Nature Reserve, Xishuangbanna, into three main production systems

		Cluster		
Trait	Unit	1	2	3
Has buffalo (yes)	% of HH	100	0	0
Has sow (yes)	% of HH	86	0	92
Corn area	ha	0.49	0.09	0.19
Rubber area	ha	0.54	2.05	1.81
Tea area	ha	0.52	0.1	0.7
Major agricultural activities		Buffalo, pig, corn	Rubber	Pig, rubber

Clusters were named after their major agricultural activities (Table 1). Simple descriptive statistics and ANOVA followed by Tamhane post hoc testing (conservative pair-wise comparisons test based on *t* test; SPSS 2007) were used to compare the final farm clusters; significance was declared at *P* < 0.05.

## Results

### General farm characteristics

Three main production systems were identified, livestock-corn-based (LB), rubber-based (RB), and pig-based (PB) farms, reassembling 41.3 %, 38.6 %, and 20.1 % of the 184 HHs (Table 2). Farms were 0.8 to 3.5 h motorbike drive away from town. Land allocated per farm was slightly less than 3 ha. Han, Dai, Hani, and Lahu were the dominant ethnic groups in the area. All HHs were headed by an adult male and consisted of one to nine individuals. About 50 % of household members had finished primary school (Table 3). In 8 % of the HHs, at least one person was member of the Communist Party of China.

**Table 2:** Overall characteristics of smallholder farm households (HH) in the Naban River National Nature Reserve, Xishuangbanna, and cropland area as well as numbers of mammalian livestock of the three main farming systems in the area

	Altitude^a^		Ethnic group^b^		Crops^c^			Livestock^c, d^		
Farming system	% of HH		% of HH		Area (ha)		SD	TLU (n)		SD
All (*n* = 184)	Lowland	48.9	Han	21.2	Rubber	1.38	1.675	Buffaloes	0.85	1.287
	Midland	22.8	Dai	19.6	Corn	0.28	0.332	Pigs	0.36	0.301
	Highland	28.3	Lahu	39.7	Tea	0.39	0.591	Sows	0.06	0.065
Livestock-corn based LB (*n* = 76)	Lowland	22.4	Han	19.7	Rubber	0.55^a,b^	0.940	Buffaloes	2.03^a,b^	1.275
	Midland	26.3	Dai	2.6	Corn	0.49^a,b^	0.375	Pigs	0.93^a^	0.665
	Highland	51.3	Lahu	57.9	Tea	0.52^a^	0.531	Sows	0.19^a^	0.119
			Hani	19.7						
Rubber-based RB (*n* = 71)	Lowland	84.5	Han	12.7	Rubber	2.05^a^	1.901	Buffaloes	0.04^a^	0.203
	Midland	9.9	Dai	46.5	Corn	0.09^a,c^	0.155	Pigs	0.39^a,c^	0.360
	Highland	5.6	Lahu	29.6	Tea	0.1^a,c^	0.216	Sows	0^a,c^	0.000
			Hani	8.5						
Pig-based PB (*n* = 37)	Lowland	35.1	Han	40.5	Rubber	1.81^b^	1.683	Buffaloes	0^b^	0.000
	Midland	40.5	Dai	2.7	Corn	0.19^b,c^	0.220	Pigs	0.96^c^	0.554
	Highland	24.3	Lahu	21.6	Tea	0.69^c^	0.890	Sows	0.21^c^	0.100
			Hani	32.4						

*SD* standard deviation, *TLU* tropical livestock unit

^a^Altitude—lowland = 400–800 m a.s.l.; midland, 801–1,200 m a.s.l.; highland > 1,200 m a.s.l.

^b^Ethnic group: difference to 100 % is due to some more minor ethnic groups

^c^Superscript letters indicate statistical differences between group means (*P* < 0.05): *a* LB *versus* RB; *b* LB *versus* PB; *c* PB *versus* RB

^d^Tropical livestock unit, equivalent of an animal of 250 kg live weight (buffalo = 1.2 TLU; pig/sow = 0.2 TLU)

**Table 3:** Selected socioeconomic characteristics of smallholder pig producers in the Naban River National Nature Reserve, Xishuangbanna

	Distance to town^**a**^		Literacy rate^b^	Household size		Non-farm income^c^	
Farming system	Hours	SD	% of HH	Persons	SD	% of HH	Range
All (*n* = 184)	1.9	0.75	44	4.6	1.97	15	1,800–50,000
Livestock-corn-based, LB (*n* = 76)	2.3	1.92	38	4.9	1.45	11	2,400–12,000
Rubber-based, RB (*n* = 71)	1.6	0.70	48	4.5	1.59	17	7,400–50,000
Pig-based, PB (*n* = 37)	2.2	0.57	54	5.2	1.69	24	1,800–24,000

^**a**^Unit: Average driving hours by motorbike during dry season from village center to the closest market in town

^b^The percentage of household members who finished primary school education

^c^Numbers were taken as provided during interviews and are not based on actual calculations, expressed in Chinese RMB (10 RMB ~ 1 EUR)

Rubber, corn, and tea were found in all farming systems, but rubber dominated RB and PB farms, while tea was mainly found in PB and LB farms. Chicken was prevalent on literally each farm, pigs in almost 90 % of HHs. Sows, however, were only reported from PB and LB farms (Table 4). Slightly less than half of all interviewed HHs were keeping one or more buffaloes. Animal manure was used by 77.2 % of all HHs to fertilize vegetable gardens (54.2 %), rice fields (47.2 %), rubber fields (38.0 %), and to feed biogas fermenters (38.7 %) providing light and fuel on the farm.

**Table 4:** Characteristics of pig management in smallholder pig farms of Naban River National Nature Reserve, Xishuangbanna

	Sows per HH		Free range period		Popularity of pig feed		Utilization of pigs	
			Months year^−1^					
Farming system	Average	% HH	Mean	SD	Component	% of HH	Utilization	% of Pigs
All (*n* = 184)	0.30	53	1.9	1.23			Self-consumed	71
							Sold	29
Livestock-corn-based, LB (*n* = 76)	0.95	86	1.9	0.43	Banana trunk	98	Self-consumed	60
					Corn	95	Sold	20
					Meal leftovers	87	Gifted	20
					Commercial feed	7		
Rubber-based, RB (*n* = 71)	0	100	1.2	0.38	Banana trunk	73	Self-consumed	57
					Rice bran	86	Sold	43
					Corn	79		
					Commercial feed	30		
Pig-based PB, (*n* = 37)	1.10	92	2.0	1.10	Banana trunk	87	Self-consumed	75
					Corn	97	Sold	25
					Meal leftovers	89		
					Commercial feed	19		

Pigs were fed with a variety of at least 18 different feedstuffs, whereby banana trunk, rice bran, corn, and “green plants from the forest” were the most widely used. Feed was prepared as a batch for 2–5 days and offered from this pool twice daily. Commercial feeds such as fishmeal were used by a small number of households (Table 4). The daily work of pig feeding was done by women and sometimes children, but the decision to purchase, sell, and slaughter pigs remained with the HH head. Piglets for fattening were purchased from traders or from highland farmers by 48 % of the pig keepers; the other 52 % pig-keeping HHs were breeders keeping sows. Few pig-keeping HHs (2.5 %) kept breeding boars; most sows mated randomly with boars from the village or wild boars in the forest during scavenging periods. Among the pig breeders, 30.5 % were actively choosing a mating partner for their sows; the remaining farmers relied on random mating. Local SMEP was the dominant breed, followed by crosses and exotic breeds. However, all farmers tended to call their pigs SMEP regardless of breed. In the 12 months preceding our survey, 60 % of pig-keeping HHs encountered pig diseases; in 76 % of all disease occurrences, the owners could not identify the disease or the reason of death. Governmental veterinarians assisted farmers in 65 % of disease outbreaks.

Pigs mainly are self-consumed; a smaller share is sold to eventually passing traders (Table 4), and 2.2 % of all pig farmers stated to earn their major income from pig production. According to group interviews, the main constraints of pig production were high disease-related losses and lack of labor time to prepare pig feed. For some farmers, lack of money to purchase additional sows was the first limiting factor. The majority of pig keepers were interested in intensifying pig production to stabilize their income. However, farmers who only fattened pigs were often satisfied with their production and not interested in production increase, although they stated to purchase meat for family consumption because the latter exceeded their production.

Tea and rubber cultivation were the main income sources of 44.6 % and 39.7 % of farmers, followed by rice (6.0 %), corn (5.4 %), and other minor income sources. Non-farm income from government employment, unskilled off-farm work, or shop-keeping was earned by 15 % of HHs. There was no family with more than one member earning non-farm income. High-value assets such as a motorbike or a tractor, respectively, were owned by 82 % and 60 % of all HHs.

### Livestock-corn based farms

The main characteristics of LB farms were their location at high altitudes, mainly Lahu tribe affiliation, and low literacy rate. Fifty-one percent of LB farms were found at >1,200 m a.s.l.; distance to town was highest among all groups, and in 26 % of LB farms no HH member was able to read. Popular jobs among LB farmers were in the “service industry” and as “government employees” (such as “forest protector” or “party secretary”), but a part of interviewees refused to state the origin of income.

LB fields were dominated by corn, tea, and rice cultivation; few farmers owned young rubber plantations. Buffaloes, pigs, and chicken were present in each household. Pigs were kept in wooden/bamboo or sometimes brick-made pens for most of the year. LB farms had the lowest use of commercial feed additives. At the time of interview, only 82 % owned sows but all LB pig keepers stated to generally breed their own offspring. While 71 % of pig-keeping LB farmers kept one sow, 10 % had two or more breeding sows, and 3 % kept a breeding boar. Only 20 % were actively choosing a mating partner for their sows, the other LB pig farmers let their sows mate randomly during free ranging periods. Maintaining the gene pool of SMEP by using only SMEP boars for breeding was the desire of 15 % of LB pig farmers. Although a general definition of SMEP characteristics could not be obtained from these respondents, no farmer desired to introduce exotic genes into his herd. Important traits for boars were strong (15 %) and large-scaled frame (13 %), while 80 % did not prefer any trait. Diseases in their herds had been recognized by 70 % of all LB pig keepers during the 12 months preceding the interview. Mortality ranged between 0 % and 100 %, and each farm had lost 4.6 ± 3.68 pigs due to diseases in the previous 12 months. Disease-affected pig farmers lacked knowledge to identify diseases but named anorexia (76 %), ‘no thirst for water’ (73 %), diarrhea (14 %), and fever (11 %) as signs of diseases in a multiple answer sheet. Fifty-one percent of pig keeping LB farmers stated to never sell pigs, and 66 % had not sold any pig in the past 12 months. LB farmers were very interested in expanding their pig production and stated that massive disease problems, ineffective support by governmental veterinarians and low growth rates of fattening pigs were the main limiters to expansion of pig production. Keeping more pigs was desired but limited by lack of labor and capital.

### Rubber-based farms

Rubber-based farms were the ones closest to town and on lowest altitude level. Dai were the dominant ethnic group in RB farms. The vast majority of cultivated land was occupied by rubber (69 %), followed by rice (15 %). Very few RB farmers kept buffaloes; about 50 % had pigs, and all were keeping chicken (Table 2). Pigs were predominantly kept in concrete stables with iron sheet roofs. Sixty-one of RB pig keepers exclusively used purchased feed, and 30.2 % purchased commercial feeds such as fishmeal. All RB farmers were only fattening purchased piglets and did not keep sows. Piglets were mainly obtained from commercial breeders in Simao area (app. 400 km north of Jinghong); they were large-framed (adult weight >150 kg) with phenotypic characteristics similar to the local breeds. In the 12 months preceding the survey, 28.3 % of the pig-keeping RB farms had been affected by pig diseases with an average loss of 1.9 ± 2.14 pigs. RB farmers were also not able to name the diseases but associated the symptoms ‘anorexia,’ ‘no thirst for water,’ and ‘diarrhea’ to 54 %, 46 %, and 39 % of the disease cases. Of the surviving pigs, 43 % were traded, the rest was self consumed. Pigs were mostly (60 %) sold to a ‘trader that came to the village’; the others were sold to villages near Jinghong and Jinghong suburbs; 6 % of RB farmers did also give away pigs as gift. Regression analysis between wealth (expressed in *size_of_rubber_fields*) and number of pigs (*TLU_pig*) of RB farmers revealed that pigs were evenly distributed among rich and poor RB farmers, with no clumping at farms with smaller rubber area (Pearson correlation *r* = −0.18; *P* = 0.896), indicating that wealthy farmers were not abandoning pig fattening. RB farmers explicitly rejected the idea to expand pig production or create integrated systems.

### Pig-based farms

Pig-based farms had similar distances to town as LB farms and were evenly distributed across altitude ranges. PB farms were the group with the highest percentage of HHs earning non-agricultural income. Executed jobs were ‘teacher’, ‘trader’, and ‘employment in the service industry’, but most persons refused to state the origin of their additional income.

Of the PB fields, 51 % were covered with rubber, 17 % with tea, 9 % with corn, and 8 % with rice (Table 2). No PB farmer kept buffaloes; all raised pigs. The average size of a pig herd was 4.8 ± 2.77 head. Pigs were mainly housed in wooden pens; 19 % of the farmers purchased fish meal. While half of the sow-owners let their sows mate randomly, the other half selected the boar for mating. Of the latter farmers, 76 % wished to mate their sows with a local SMEP boar, 18 % with a crossbreed local × improved boar. The rest preferred other undefined local breeds. Healthy, strong, and large-framed were the primary selection criteria for a boar. Half of the RB farmers (49 %) reported occurrence of pig diseases in their herd in the past 12 months, with an average loss of 7.1 ± 10.82 pigs. Anorexia and ‘not thirsty’ (62.5 % each) as well as ‘lack of energy’ and diarrhea (19 % each) were the most frequently recognized signs of disease. Twenty-five percent of the surviving pigs were sold, 47 % of them to traders visiting the villages; all others were sold to RB farmers. PB farmers showed interest in expanding pig production but pointed to disease risks and lack of labor for feeding pigs as main constraints to intensified production.

## Discussion and conclusions

The combination of CatPCA with SPSS two-step clustering proved to create meaningful classes and reliably allot farms to these; the three farm types were significantly different in their setup and management. Similar soundness of the combination of CatPCA with two-step-clustering to classify multi-attributed data in household studies and creating solid and meaningful household clusters were reported by Dossa et al. (2011). The three farm types were significantly different in their setup and management practices. According to Notenbaert et al. (2009) a sound exploration of smallholders’ full situations, including social, natural, and technical aspects, is required to successfully support their development.

### Socio-economic aspects

Summarizing the socio-economic characteristics of the three farm types, more poor self-subsistent farmers were living in the uplands, whereas richer farmers involved in cash-cropping and marked-oriented livestock production predominantly lived in the lowlands. Jalan and Ravallion (2002) also identified the location of a farm in China as a significant determinant for household consumption and reported the factors *montane_area* and *bad_road_access* to be major inhibitors for increasing consumption. Such areas were therefore named “geographical poverty traps”. Lahu people, described as “socially weak and uneducated” (Du 2000), are captured in such traps of geographical and cultural distance to town in the NRNN Reserve. Contrarily, Dai and Hani farmers (dominating RB and PB clusters) have strong relations to the town and often are involved in trade (Sturgeon 2010). Distance to town of those groups might not always be shorter than that of the Lahu, and rather, their cultural characteristics make the former better situated. Central and provincial government acknowledge the “periphery syndrome” of minority people in “periphery areas” and implemented measures such as minority schools, but in reality equal chances of underdeveloped tribes and areas often get stuck at local levels (Hansen 1999; Wang and Zhou 2003).

### Land cultivation

The variable *“size_of_rubber_fields”* was a major determinant for the farming systems classification in the NRNNR. Compared with rice, a 15-fold higher income per area of land is gained through rubber (Qiu 2009). Consequently, wherever possible, rubber replaces all other crops, and RB farmers underwent a change “beyond our imagination” (Qiu 2009). An increase of the rubber-based income of 438 % was calculated for some villages in the region (1998–2004; Fu et al. 2009). Strong price fluctuations and weaknesses of monoculture to disease infestations and soil degradation (Wolff and Zhang 2010), however, makes rubber planting a highly sensitive income source. Similarly, tea prices are heavily influenced by traders and financial gambling (Chinaeconomic.net 2007). A more diverse production structure could avoid impacts of heavy economic or ecological shocks for RB and tea-focused farms.

#### Livestock husbandry

Very poor rural dwellers benefit from livestock rearing (Steinfeld et al. 2006), and the combination of plant and animal production has positive effects on the economy and ecology of small farms (Devendra and Thomas 2002b; Gura 2008). Buffaloes, which have been a major asset in South-East Asian agricultural systems for decades, are losing importance in NRNNR farming. Highland farmers still utilize buffaloes on steep rice terraces where they are easier to manipulate than tractors, but they do not play a role in mid- and lowland farming anymore: Higher availability of tractors through better infrastructure and economic conditions in the mid- and lowlands decreased the value of buffaloes (Nanda and Naka 2002). Buffalo meat, when required for example for ceremonies, is nowadays purchased from town markets and highland farmers.

Similar to the buffalo, pig-keeping is no income source for RB farms any more, since only a few pigs are fattened; no offspring is produced, and often, purchased fodder is used. Demanded pork meat is also available through daily traders. Wealth, however, is not leading HHs to abandon pig husbandry (see section “Results” and “Rubber-based farms”). Strong historical bounds (Kim et al. 1994) and actual cultural structures of gifting and bribing (Millington et al. 2005) are reasons for richer people to still keep pigs. In fact, most pig deals of RB farmers were done with partners who were close to or from the city. Despite significant economic and ecological benefits from combining livestock and rubber farming (Paris 2002), incentives for integration are low compared with the earnings from rubber alone in the target area. RB farmers’ pig-keeping will therefore remain at its actual status whereby PB and LB farms will have more chances to sell pigs to richer lowland farmers in future.

### Pig husbandry

While farmers reported to have their pigs scavenge freely for almost the whole year in past times, the risk of destroying high-value cash crops nowadays forces them to keep pigs in pens. Farmers in all classes therefore need to provide all feed required. RB and PB farms tended to use more purchased fodder while LB farmers mainly utilized self-cultivated or collected feedstuffs. Feed composition is similar to that of smallholder systems all across Laos, Vietnam, and Cambodia (Loc et al. 1997; Hai and Pryor 1996; Rodríguez and Preston 1997). Current feeding practices require high labor input for fodder collection and preparation (2–3 h per day; Stür et al. 2002), and diets are often energy-rich but protein-deficient (e.g., Loc et al. 1997; Rodríguez and Preston 1997).

Lack of appropriate fodder impacts the productivity of the whole systems through higher disease susceptibility and reproductive failure of affected animals (Stür et al. 2002). Better utilization of available feeds combined with efforts to make new ones available might therefore have the strongest impact on productivity (Thomas et al. 2002). However, available literature only points to general pathways for improved feeding. Therefore, a more detailed analysis of farmers’ feeding practices, the used feedstuffs, and the seasonal patterns of nutrient supply could identify bottlenecks and suitable feeding systems for pigs in the NRNNR and adjacent areas.

The high pig mortality rates are confirmed by other studies: Pig farmers in Lao highlands were reported to often experience epidemic pig diseases that killed most pigs in a single outbreak (Stür et al. 2002). Besides lethal diseases, farmers reported high numbers of gastrointestinal helminths in slaughtered pigs, but lacked knowledge about their origin, impact, and prevention measures. Effective disease prevention is therefore another key issue for improving pig production in Xishuangbanna. Although institutional capacities exist and might be able to cope with such challenges, the flow of knowledge about animal hygiene and management to smallholders in more remote villages seems to be a bottleneck for progress.

### Pig marketing strategies

After resolving the major shortcomings of feeding and health care, the implementation of an effective marketing strategy for the SMEP would help farmers to increase their income and expand their shock-absorbing capability (Perry et al. 2002). Preference of urban dwellers to purchase local products on local markets are observed in China’s Megacities (Goldman et al. 1999, 2000) and also in Jinghong, and were confirmed for SMEP products as well (Qian 2009). Producers additionally could take advantage of the newly emerging countrywide demand for ecologically produced food (Hoering 2009, 2010a; FAO 2010; Hoering and Gura 2010) and provide eco-products through supermarkets (Paull 2008) or direct sales (Anonymous 2008; Neo and Chen 2009).

The recent development indicates that there is a realistic chance for NRNNR pig farmers to market their products. While RB farms may lack incentive to take up to pork marketing, PB and especially LB farmers could significantly improve their livelihoods through this new marketing opportunity. However, access to the market and proper information is a big challenge to them (IFPRI 2005). The frequent “random farm gate selling to mobile traders” points to the farmers’ disadvantage vis-à-vis middlemen, and Samkol et al. (2006) described such farmers as rather being “price takers” than “price makers”. The formation of cooperatives and similar groups, or avoidance of middlemen by establishing direct business relations with retailers would strengthen small-scale farmers. However, such incentives are far beyond the capacity of geographically and culturally remote pig producers and require substantial external support.

## Footnotes

1
http://www.unesco.org/new/en/natural-sciences/environment/ecological-sciences/man-and-biosphere-programme/
